# Impact of Maternal Euthyroid Autoimmune Thyroiditis on Minipuberty in Female Offspring

**DOI:** 10.3390/nu18121841

**Published:** 2026-06-07

**Authors:** Karolina Kowalcze, Johannes Ott, Giovanni Cangelosi, Joanna Kula-Gradzik, Andrea Deledda, Robert Krysiak

**Affiliations:** 1 Department of Pediatrics in Bytom, Faculty of Health Sciences in Katowice, Medical University of Silesia, Stefana Batorego 15, 41-902 Bytom, Poland; jkula-gradzik@sum.edu.pl; 2 Department of Pathophysiology, Faculty of Medicine, Academy of Silesia, Rolna 43, 40-555 Katowice, Poland; 3 Clinical Division of Gynecologic Endocrinology and Reproductive Medicine, Department of Obstetrics and Gynecology, Medical University of Vienna, 1090 Vienna, Austria; johannes.ott@meduniwien.ac.at; 4 School of Pharmacy, Experimental Medicine and “Stefania Scuri” Public Health Department, University of Camerino, 62032 Camerino, Italy; giovanni01.cangelosi@unicam.it; 5 Endocrinology and Obesity Unit, Department of Medical Sciences and Public Health, University of Cagliari, 09124 Cagliari, Italy; andredele@tiscali.it; 6 Department of Internal Medicine and Clinical Pharmacology, Medical University of Silesia, Medyków 18, 40-752 Katowice, Poland; rkrysiak@sum.edu.pl

**Keywords:** genital organs, hypothalamic–pituitary–ovarian axis, infancy, pregnancy complications, saliva, thyroid autoimmunity, women’s health

## Abstract

Background/Objectives: Minipuberty is a transient activation of the hypothalamic–pituitary–gonadal axis in infancy that contributes to the postnatal development of sexual organs. Its course has been shown to be influenced by maternal hypothyroidism. This study aimed to evaluate the reproductive axis and genital development in infant girls born to women with euthyroid autoimmune thyroiditis. Methods: The study involved three groups of infants: two groups were daughters of euthyroid women with autoimmune thyroiditis, while the third group (control) consisted of daughters of women without thyroid disease during pregnancy. Half of the mothers with thyroiditis received additional vitamin D and selenium supplementation during pregnancy, whereas the other half did not. During the first 18 months of life, periodic assessments were conducted of gonadotropin concentrations in urine, as well as salivary levels of estradiol, progesterone, testosterone, androstenedione, and DHEA-S. Additionally, ovarian volume, uterine length, and breast diameter were measured in the infants. Results: Daughters of women with autoimmune thyroiditis who did not receive supplementation during pregnancy exhibited lower levels of LH, estradiol, and progesterone, as well as a more rapid decline in LH and estradiol to below detectable levels, compared with daughters of healthy women. These hormonal differences were accompanied by smaller uterine length and breast diameter in this group. No differences were observed between the offspring of non-supplemented women with thyroiditis and daughters of healthy women regarding the levels of other hormones or ovarian volume. The dynamics of all assessed hormone levels and organ measurements did not differ between daughters of euthyroid women with thyroiditis who received vitamin D and selenium supplementation and daughters of healthy women. LH and progesterone levels showed inverse correlations with anti-thyroid peroxidase antibody titers, whereas uterine and breast dimensions positively correlated with estradiol levels. Conclusions: These findings suggest that maternal euthyroid autoimmune thyroiditis can affect the progression of female minipuberty, while supplementation with vitamin D and selenium during pregnancy may mitigate this effect.

## 1. Introduction

The concept of minipuberty refers to a transient phase of activation of the hypothalamic–pituitary–gonadal axis that occurs in early childhood and is characterized by a sex-specific course [1,2,3,4,5]. In girls, minipuberty appears to follow a biphasic pattern, with the first peak in gonadotropin and estradiol concentrations occurring in the third to fourth week of life and a second peak between weeks 15 and 24 of life [6]. This contrasts with the single-peak pattern of secretion of these hormones observed in boys [1]. Moreover, throughout the entire period of minipuberty in girls, follicle-stimulating hormone (FSH) levels exceed luteinizing hormone (LH) levels, and estradiol concentrations are higher than testosterone concentrations (whereas the opposite pattern of gonadotropin and sex steroid secretion is observed in boys) [1,2]. A third important sex-related difference concerns the duration of minipuberty, which is longer in girls [4]. Detectable estradiol concentrations have been reported even during the second year of life, and the period during which FSH can be detected may extend into the third to fourth year of life, whereas in boys, activation of the hypothalamic–pituitary–testicular axis is observed exclusively during the first year of life [7]. Differences in gonadotropin and sex steroid secretion appear to have significant physiological relevance. In girls, a normal course of minipuberty determines changes in the size of the ovaries, uterus, and breasts in early childhood and may also contribute to their enlargement several years later during puberty [3]. Variations in the course of minipuberty may additionally play a role in sex-specific differences in growth dynamics and fat distribution [4]. Finally, there is a view that a normal course of minipuberty is essential for the proper development of cognitive and sensory functions [7].

The normal course of minipuberty may be disrupted in the presence of complications, particularly prematurity and low birth weight relative to gestational age. Prematurely born female infants exhibited higher serum concentrations of estradiol and inhibin B [8] as well as elevated urinary levels of estradiol and FSH [9,10] compared with full-term infants. These biochemical differences between preterm and term infants were accompanied by differences in the number of antral follicles per ovary, breast diameter growth, and uterine length [9,10]. Furthermore, female infants born small for gestational age demonstrated higher FSH and sex hormone levels than those born appropriate for gestational age [11], and estradiol concentrations in the former group were inversely correlated with weight for gestational age [8]. This suggests that the dynamics of hypothalamic–pituitary–gonadal axis activity during infancy can be affected by a nonphysiological course of pregnancy. This assumption has been supported by studies conducted by our research group [12,13,14]. An abnormal course of female minipuberty was observed in cases of untreated or inadequately treated maternal hypothyroidism and gestational diabetes, as well as in states of vitamin D deficiency; in each of these conditions, the observed disturbances correlated with the degree of hormonal and metabolic imbalance. Notably, the effects of these conditions differed substantially with respect to the direction of the observed changes, peak values, and periods of detectability [12,13,14]. This argues against interpreting the observed alterations as a consequence of a nonspecific fetal response to adverse changes in the maternal environment that is similar across various pathophysiological conditions.

The association between maternal hypothyroidism and an abnormal course of minipuberty has been demonstrated in both sexes and, in each case, was characterized by reduced activity of the hypothalamic–pituitary–gonadal axis [12,15]. However, these studies did not allow for the unequivocal exclusion of the possibility that the observed changes were at least partially related to the presence of autoimmune thyroiditis, also known as Hashimoto’s disease, which is by far the most common cause of hypothyroidism in developed countries [16,17]. In affected mothers, this condition predisposes to the aforementioned states associated with an altered course of minipuberty in their offspring, namely preterm delivery and the birth of a small-for-gestational-age infant [18,19]. It remains unclear whether these complications result from systemic inflammation or thyroid hormone deficiency. Moreover, despite the lack of an established cause-and-effect relationship, a documented association exists between Hashimoto’s disease and vitamin D deficiency [20], which—if present during pregnancy—has been shown to influence the course of minipuberty in female offspring [14]. Support for the hypothesis that autoimmune mechanisms may underlie alterations in the dynamics of the hypothalamic–pituitary–ovarian axis in mothers with hypothyroidism is further provided by the observation that delayed pubertal onset and slower pubertal progression are statistically more frequent in children with other autoimmune diseases, including juvenile idiopathic arthritis, type 1 diabetes mellitus, celiac disease, and inflammatory bowel disease [21,22,23,24].

To date, no study has evaluated the impact of autoimmune thyroiditis on the course of minipuberty, despite the high prevalence of this condition among women of reproductive age [16,17]. Therefore, the aim of our study was to investigate whether the presence of untreated Hashimoto’s disease in the euthyroid stage affects the dynamics of hypothalamic–pituitary–ovarian axis activity and genital organ size, as well as to assess the impact of exogenous vitamin D and selenium supplementation on potential alterations in the course of minipuberty.

## 2. Materials and Methods

The present study was a prospective, matched cohort study conducted from May 2022 through September 2025. The research protocol was approved by the Bioethical Committee of the Medical University of Silesia, and all procedures adhered to the ethical principles outlined in the 1964 Declaration of Helsinki. Before participation, parents or legal guardians received comprehensive information regarding the study’s objectives and potential risks, and written informed consent was obtained for each child.

### 2.1. Participants

Ninety-six apparently healthy infant girls, born within the four weeks prior to study initiation, were enrolled. Both the infants and their mothers resided in the Silesian Voivodeship, a highly urbanized region in southern Poland. Participants and their parents were referred to the study center by physicians collaborating with the research team, who provided medical care to both mothers and newborns. The study was designed to include three groups: two groups comprised daughters of mothers with euthyroid autoimmune thyroiditis during pregnancy, while the third group served as a control and included offspring of healthy women without documented pathology, including thyroid disorders.

The criterion for classifying a child as the daughter of a woman with autoimmune thyroiditis in the euthyroid state (groups A and B) required the simultaneous fulfillment of the following conditions: (1) thyroid peroxidase antibody (TPOAb) titers exceeding three times the upper limit of normal; (2) TSH concentrations, and—if measured—free thyroid hormone levels within the reference range, based on American Thyroid Association recommendations for pregnancy [25]; and (3) a typical ultrasonographic appearance of autoimmune thyroiditis in an examination performed during pregnancy. For TPOAb antibodies, titers were required to exceed the established threshold at least twice: at least once in the year preceding confirmation of pregnancy and at least once during pregnancy. For TSH, at least four measurements within the normal range were required: at least one within the six months preceding pregnancy and at least three during pregnancy, including at least one in the first trimester.

Thyroid disease in mothers without thyroid pathology (group C) was excluded based on medical history, TPOAb and TSH results, and the absence of abnormalities on ultrasound. Normal TPOAb titers (and, if tested, thyroglobulin antibodies [TgAb]) had to be documented in at least one measurement either during pregnancy or within the six months preceding conception. Normal TSH levels were defined as at least two measurements within the reference range, including at least one during pregnancy, falling between the lower reference limit (reduced by 0.4 mU/L in the first trimester) and 4.0 mU/L. The absence of thyroid abnormalities was determined based on the mother’s ultrasound performed during pregnancy, or, if unavailable, at the first visit with the child.

Infants were excluded if diagnosed with major congenital anomalies, cryptorchidism, congenital infections, genetic syndromes, or perinatal asphyxia; if they had any chronic disorder; if they were premature (born at or before 36 weeks of gestation); or if they required prolonged pharmacological treatment, excluding vitamin D supplementation. Maternal exclusion criteria encompassed discrepant laboratory findings during pregnancy, isolated hypothyroxinemia (defined as free thyroxine levels below 10.0 pmol/L with normal TSH), gestational diabetes or preexisting type 1 or type 2 diabetes, mental health conditions, other chronic medical conditions during pregnancy, or pharmacological therapy during gestation (or lactation in breastfeeding women) lasting more than 10 days, except for vitamin or micronutrient supplementation intended for pregnant or breastfeeding women. Additional maternal exclusions included pregnancy complications requiring urgent hospitalization, as well as a history of alcohol or substance abuse.

Group A included daughters of women with euthyroid autoimmune thyroiditis who did not receive additional vitamin D or selenium supplementation during pregnancy. Group B consisted of daughters of women with this disorder who, during pregnancy, received supplements containing vitamin D and selenium at daily doses exceeding 37.5 µg and 55 µg, respectively. These values correspond to the doses of vitamin D and selenium contained in prenatal preparations available on the Polish market and appear to be safe during this stage of life. Moreover, in the authors’ own observational data, their use among women with autoimmune thyroiditis was associated with lower titers of thyroid antibodies during pregnancy compared with those who did not receive such supplementation. The minimum duration of vitamin D and selenium supplementation during pregnancy in this group was six months. If the mother took lower doses or supplemented for a shorter period, the child was deemed ineligible for inclusion in the study.

The estimated sample size was 25 participants per group to detect a 25% difference in salivary estradiol, assuming a type I error rate of 5% and 80% statistical power. However, the initial group size was increased to 32 children per group to account for expected attrition due to the study’s long duration and strict eligibility criteria.

Recruitment aimed to maintain comparable numbers of children across seasons to minimize potential seasonal influences on hormonal measurements. Each study group was selected from a larger pool of potential participants to generate three populations matched for age, parity, gestational age at birth, and mean maternal TSH concentration during pregnancy. Additionally, for the groups of children born to mothers with autoimmune thyroiditis, participants were matched for comparable TPOAb titers in the year preceding conception.

### 2.2. Study Design

The study lasted eighteen months, with the intervals between consecutive visits depending on the child’s age. Between the first and sixth month of life, visits occurred monthly; during the second half of the first year, they took place every two months; and between the twelfth and eighteenth month, visits were scheduled at three-month intervals. Each visit included four components, the first two of which were mandatory: (1) collection of a medical history regarding the child from a parent or guardian, (2) a detailed clinical assessment of the child, (3) anthropometric measurements, and (4) collection of biological samples, including urine and saliva, for biochemical analysis. The medical history included a subjective assessment of the child’s overall health, development since the previous visit, any illnesses or hospitalizations, adherence to the vaccination schedule, and, if applicable, medications administered. The detailed clinical assessment comprised, in addition to the physical examination, the interpretation of laboratory and imaging tests performed during that period. If the evaluation indicated that the child was healthy and had not received pharmacological treatment for at least ten days prior to the visit, anthropometric measurements were taken and biological samples were collected. Administration of mandatory vaccinations during this period did not constitute a contraindication to completing the full visit protocol. Final analyses included only those children who attended at least eight of the eleven scheduled visits, with a minimum of five visits during the first six months of life, at least two visits during the second six months, and at least one visit during the third six months.

Dietary intake of vitamin D and selenium during pregnancy was assessed at the first study visit. For women who kept dietary records during pregnancy, intake estimates were calculated based on analyses of these records. Participants who did not maintain dietary records completed a structured questionnaire designed to assess the amount and frequency of consumption of commonly eaten Polish dishes during the final eight weeks of pregnancy. Consumption frequency was categorized as daily, 5–6 times per week, 3–4 times per week, 1–2 times per week, less frequently, or never. Mean daily intake of both micronutrients from dietary sources was estimated using reported food types, composition, and portion sizes, in conjunction with standardized food composition and nutrition tables [26].

### 2.3. Anthropometric Measurements

Crown-to-heel length was measured to the nearest millimeter with the infant in the supine position using a portable infantometer (Seca, Hamburg, Germany). Body weight was recorded to the nearest 10 g using a digital infant scale (Seca 834, Hamburg, Germany). Infants were weighed unclothed and without diapers. Weight-for-length was calculated by dividing weight by length and then converted to percentiles according to World Health Organization standards. Head circumference was measured to the nearest millimeter using a flexible, non-stretch tape, aligned over the glabella and the posterior occipital protuberance.

Ovarian volume and uterine length were assessed using ultrasonography with a high-resolution transducer operating at a frequency range of 5–12 MHz. Examinations were preferably performed with the urinary bladder filled, allowing it to serve as an acoustic window. Each measurement was performed three times, and the results were averaged. Ovarian volume was calculated using the prolate ellipsoid formula: volume = longitudinal diameter (cm) × transverse diameter (cm) × anteroposterior diameter (cm) × 0.52. When both ovaries were visualized, the calculated ovarian volume was the mean of the right and left ovary measurements. If only one ovary was visualized, its measured volume was taken as the ovarian volume. Uterine length was measured from the top of the fundus to the external cervical opening at the greatest length of the midsagittal plane. The mammary gland was examined following the protocol described by Henriksen et al. [27]. Breast tissue was first identified by palpation as a firm subcutaneous disc, and its diameter was measured to the nearest millimeter using a caliper (Insize Europe, Zamudio, Spain). Measurements below 3 mm, corresponding to the nipple diameter, were deemed unmeasurable and recorded as 1 mm. Each gland was measured three times, and the mean was calculated for each; the final value was taken as the average of the measurements from the right and left breast.

### 2.4. Laboratory Assays

Urine and saliva samples were collected in the morning hours (7:00–9:00 a.m.) under conditions ensuring the child’s comfort. Urine was collected using specially designated collection bags. The bag was applied by the child’s parents or caregivers and subsequently removed and secured by a member of the research team. Prior to application, the child’s genital area was thoroughly cleansed and then dried with a wipe. After removal of the protective paper from the adhesive surface, the upper portion of the bag was affixed to the skin above the genital area, the lower portion was secured posteriorly, behind the vagina, and the sides were attached along each leg. Care was taken to ensure that the bag adhered firmly to the skin, thereby preventing urine leakage and contamination. Following urination, the bag was incised, and the urine was transferred into a designated specimen container. Saliva collection was conducted after a minimum interval of one hour following the last feeding to minimize contamination with food particles and to avoid any potential effects of feeding on the secretion of the hormones being assessed in saliva. Saliva was obtained using sterile, single-use 2 mL syringes. The syringe was carefully placed in the child’s oral cavity, and saliva was aspirated by gently retracting the plunger. The abundant saliva production typical of this stage of life, the experience of the individual performing the procedure, and the short duration of the procedure (40–60 s) contributed to its excellent tolerability and absence of stress in the children. After thawing, the samples were centrifuged again, and LH and FSH were measured in urine, while estradiol, progesterone, testosterone, androstenedione, and dehydroepiandrosterone sulfate (DHEA-S) were measured in saliva using the enzyme-linked immunosorbent assay (ELISA) method [12,13]. To enhance the reliability of the results, all measurements were performed in duplicate. Due to the influence of urine concentration on hormone assay results, the obtained gonadotropin concentrations were normalized by dividing by the urinary creatinine concentration [28]. Kits were purchased from Abbott Laboratories (Green Oaks, IL, USA) for FSH and LH; from Salimetrics (Carlsbad, CA, USA) for estradiol, androstenedione, and DHEA-S; from BioVendor R&D (Brno, Czech Republic) for progesterone; and from DiaMetra (Perugia, Italy) for testosterone. Catalog numbers were as follows: 2P40 (LH), 7K75 (FSH), 1–3702 (estradiol), RTC016R (progesterone), DKO021 (testosterone), 1–2902 (androstenedione), and 1–1252 (DHEA-S). Intra- and inter-assay coefficients of variation were 3.5% and 4.9% for LH, 3.8% and 5.2% for FSH, 5.3% and 6.0% for estradiol, 5.1% and 6.8% for progesterone, 6.0% and 7.2% for testosterone, 6.2% and 8.5% for androstenedione, and 6.1% and 7.5% for DHEA-S. Limits of detection (LOD) were 0.1 U/L for LH and FSH, 4 pmol/L for estradiol, 16 pmol/L for progesterone, 10 pmol/L for testosterone, 18 pmol/L for androstenedione, and 100 nmol/L for DHEA-S. Recovery ranged between 87% and 114%, depending on the analyte.

### 2.5. Statistical Analysis

When at least one result exceeded the detection threshold, values below the limit of detection were assigned the limit of detection value; statistical analyses were not performed if all results were below this threshold. All numerical variables were log-transformed to satisfy the assumptions of normality and homoscedasticity. Intergroup comparisons were conducted using one-way analysis of variance (ANOVA), with subsequent multiple comparisons performed using Bonferroni’s post hoc test. Repeated-measures ANOVA was employed for within-group comparisons, followed by Tukey’s post hoc test for pairwise differences. Differences in qualitative variables were evaluated using the chi-square test. Relationships between variables were assessed via Pearson’s correlation for continuous pairs, the phi coefficient for continuous–categorical pairs, and point-biserial correlation for categorical pairs. Statistical significance was defined as two-tailed *p*-values < 0.05 after correction for multiple comparisons.

## 3. Results

**Figure 1:**
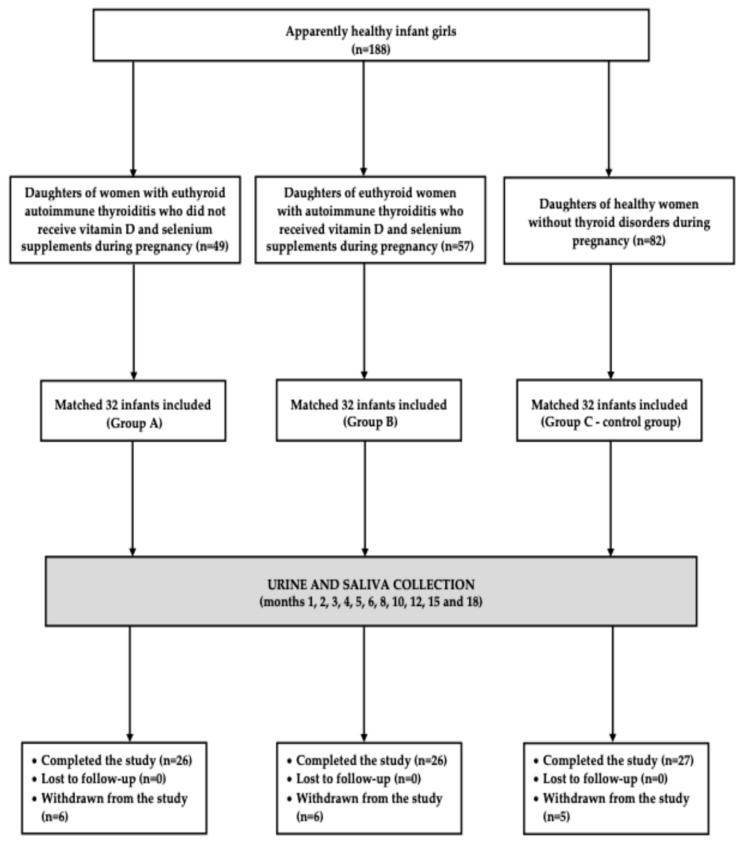
The flow of participants throughout the study phases.

The most common reason for premature withdrawal from the study was recurrent infections, primarily of the upper respiratory tract, which made it impossible to collect biological samples at the required number of time points. This situation affected a total of 14 infants: four each from groups A and C, and five from group B. In the case of two children (from groups A and B), withdrawal from the study resulted from the identification of maternal conditions during pregnancy—specifically, pregnancy-induced hypertension and gestational diabetes, respectively. In the former case, the mother had been receiving chronic antihypertensive treatment with methyldopa. The investigators were informed of these conditions only during the course of the study. For one infant (from group A), withdrawal was due to a diagnosis of gastroesophageal reflux disease and the resulting need for medical treatment. Additionally, one girl from group C discontinued participation at the parents’ request, citing family-related reasons. Overall, 79 infants completed the study, representing 82.3% of the enrolled study population (Figure 1).

Analysis of mothers of female participants showed that the study groups were comparable in age, education, employment and occupation type, parity, smoking status, body mass index, and systolic and diastolic blood pressure. The groups showed differences in mean vitamin D and selenium intake, with higher values observed in group B compared to the other groups, and no differences between groups A and C (Table 1). No mother had comorbidities or used medications that could have affected the assessed hormone levels. The average duration of vitamin D and selenium supplementation in group B was 31 ± 3 weeks.

Prior to pregnancy, thyroid antibody titers were comparable between groups A and B, with both groups exhibiting higher levels than group C. The evaluated groups did not differ in terms of TSH, free thyroxine, SPINA-GT and SPINA-GD before pregnancy. In women with autoimmune thyroiditis, TPOAb titers were higher before pregnancy relative to those measured during pregnancy. During pregnancy, thyroid antibody titers were lower in group B than in group A. No significant changes were observed in TSH concentrations, free thyroid hormone levels, or Jostel’s index between the pre-pregnancy period and pregnancy in either group A or B. In group B, SPINA-GT and SPINA-GD values increased during pregnancy relative to the pre-pregnancy period, whereas this pattern was not observed in group A. During pregnancy, both SPINA parameters differed significantly between the two groups (Table 2).

No significant differences were observed between the study groups in gestational age at delivery, birth order, length, weight, weight-for-length percentile, head circumference, breastfeeding status, or daily intake of vitamin D and selenium from both dietary sources and supplements (Table 3). Similarly, the mean duration of both exclusive breastfeeding and breastfeeding combined with complementary feeding did not differ between the groups.

**Figure 2:**
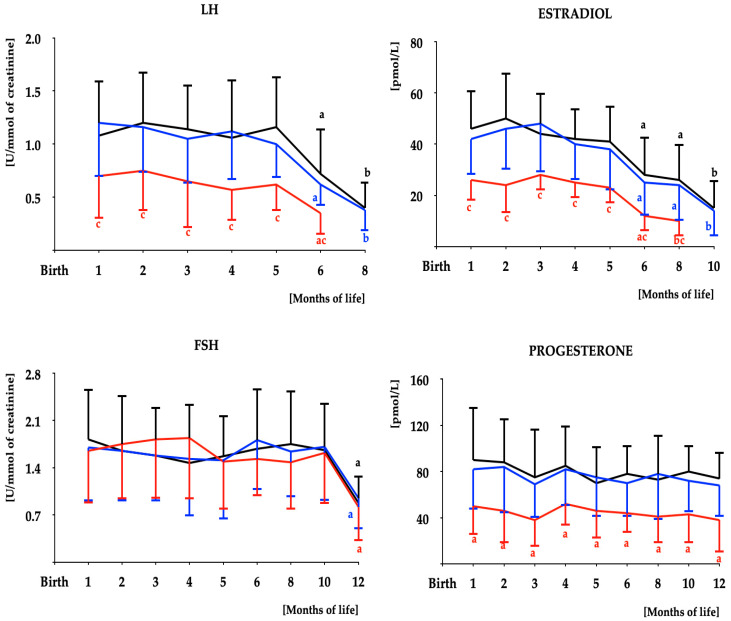
Urinary gonadotropin levels and salivary estradiol and progesterone levels in study participants. Data are expressed as mean ± standard deviation. LH was undetectable in urine from month 8 until the end of the study in group A and from month 10 until the end of the study in groups B and C. FSH was undetectable in urine from month 15 until the end of the study. Estradiol was undetectable in saliva from month 10 in group A and from month 12 in groups B and C until the end of the study. Progesterone was undetectable in saliva from month 15 until the end of the study. Group A (red line): Daughters of women with euthyroid autoimmune thyroiditis who did not receive vitamin D or selenium supplementation during pregnancy; Group B (blue line): Daughters of euthyroid women with autoimmune thyroiditis who received vitamin D and selenium supplementation during pregnancy; Group C (black line): Daughters of healthy women without thyroid disorders during pregnancy. Statistical annotations: LH—ᵃ *p* < 0.05 versus months 1–5 within the same study group, ᵇ *p* < 0.05 versus months 1–6 within the same study group, ᶜ *p* < 0.05 versus the corresponding time point in the other groups; FSH—ᵃ *p* < 0.05 versus months 1–10 within the same study group; estradiol—ᵃ *p* < 0.05 versus months 1–5 within the same study group, ᵇ *p* < 0.05 versus months 1–8 within the same study group, ᶜ *p* < 0.05 versus the corresponding time point in the other groups; progesterone—ᵃ *p* < 0.05 versus the corresponding time point in the other groups.

Urinary LH levels were detectable from month 1 through month 6 in group 1 and through month 8 in groups 2 and 3. Across all study groups, LH concentrations remained stable between months 1 and 5 and declined thereafter. Throughout the detection period, urinary LH concentrations were lower in group 1 than in the other two groups. FSH was detectable in urine throughout the first 12 months of life. Levels remained stable from month 1 to month 10 and then declined. No differences were observed between the groups at corresponding time points. Estradiol was detectable in saliva from month 1 through month 8 in group A and through month 10 in groups B and C. In each group, estradiol levels remained stable during the first five months and decreased thereafter. During the first ten months, salivary estradiol concentrations were lowest in group A and did not differ between groups B and C. Salivary progesterone remained detectable throughout the first year of life in all study groups, with concentrations remaining stable over time. Levels in group A were lower than in the other groups, whereas groups B and C were comparable (Figure 2).

The patterns of change in LH, FSH, estradiol, and progesterone concentrations were similar after excluding offspring of 14 women with a mean pregnancy TSH level exceeding 2.5 mU/L from the analysis (Supplementary Figure S1).

**Figure 3:**
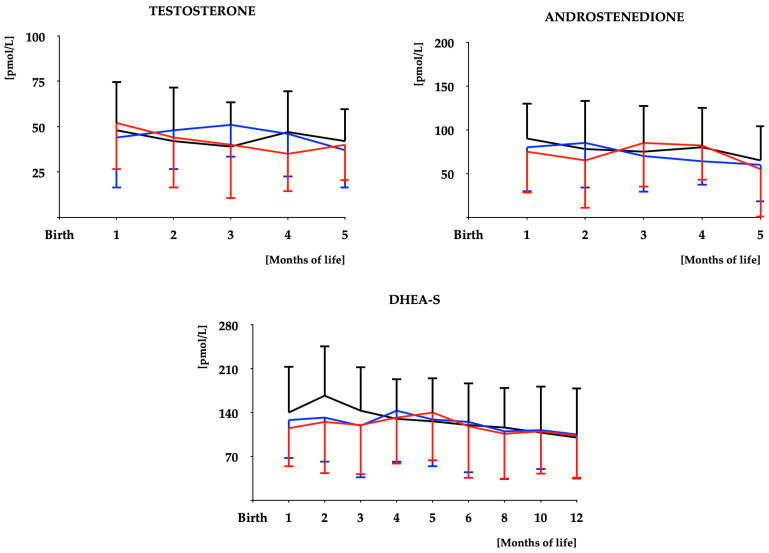
Salivary androgen levels in study participants. Data are expressed as mean ± standard deviation. Testosterone and androstenedione were undetectable in saliva from month 6 until the end of the study, whereas DHEA-S was undetectable from month 15 until the end of the study. Group A (red line): Daughters of women with euthyroid autoimmune thyroiditis who did not receive vitamin D or selenium supplementation during pregnancy; Group B (blue line): Daughters of euthyroid women with autoimmune thyroiditis who received vitamin D and selenium supplementation during pregnancy; Group C (black line): Daughters of healthy women without thyroid disorders during pregnancy.

Salivary concentrations of the remaining steroid hormones were detectable from month 1 to month 5 for testosterone and androstenedione and from month 1 to month 12 for DHEA-S. Both hormone levels and detection periods were consistent across all study groups and remained stable throughout the study period (Figure 3).

**Figure 4:**
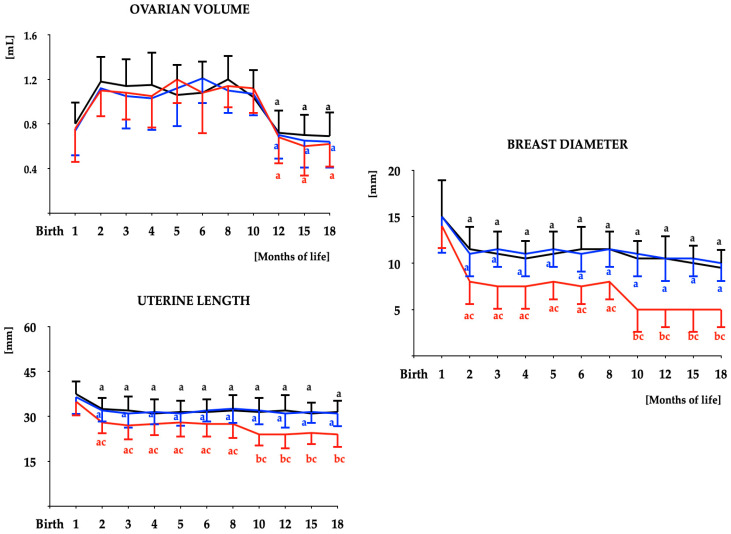
Ovarian, uterine, and breast size in study participants. Data are expressed as mean ± standard deviation. Group A (red line): Daughters of women with euthyroid autoimmune thyroiditis who did not receive vitamin D or selenium supplementation during pregnancy; Group B (blue line): Daughters of euthyroid women with autoimmune thyroiditis who received vitamin D and selenium supplementation during pregnancy; Group C (black line): Daughters of healthy women without thyroid disorders during pregnancy. Statistical annotations: ovarian volume—ᵃ *p* < 0.05 versus months 2–10 within the same study group; uterine length—ᵃ *p* < 0.05 versus month 1 within the same study group, ᵇ *p* < 0.05 versus months 1–8 within the same study group, ᶜ *p* < 0.05 versus the corresponding time point in the other groups; breast diameter: ᵃ *p* < 0.05 versus month 1 within the same study group, ᵇ *p* < 0.05 versus months 1–8 within the same study group, ᶜ *p* < 0.05 versus the corresponding time point in the other groups.

In all groups, ovarian volume increased from month 1 to month 2, remained stable between months 2 and 10, and decreased thereafter. Over the study period, there were no differences in ovarian volume between the study groups at corresponding time points. Uterine length decreased between month 1 and month 2 in all study groups. In group A, it remained stable from month 2 to month 8 and then declined. In groups B and C, it remained stable from month 2 until the end of the study. Between months 2 and 18, uterine length was smaller in group A than in the other groups, with no differences between groups B and C at any time point. All study groups experienced a decrease in breast diameter between month 1 and month 2. In group A, measurements remained consistent from month 2 through month 8 before declining. In groups B and C, breast diameter remained stable from month 2 until the end of the study. Between months 2 and 18, group A had a smaller mean breast diameter than the other groups, whereas groups B and C did not differ at any time point (Figure 4).

During the study period, all groups showed positive correlations between estradiol levels and LH concentrations, uterine length, and breast diameter, as well as between FSH levels and ovarian volume. Additionally, in groups A and B, negative correlations were observed between gestational TPOAb titers and maternal vitamin D and selenium intake, SPINA-GT values during pregnancy, and infant LH and progesterone levels. In both groups, TPOAb titers positively correlated with mean gestational TSH levels (Table 4).

## 4. Discussion

The most significant finding of the present study was the demonstration that the presence of autoimmune thyroiditis alters the course of minipuberty in the daughters of women affected by this condition, despite the fact that during pregnancy and throughout the six months preceding conception, serum concentrations of hormones of the hypothalamic–pituitary–thyroid axis remained within normal reference ranges. The applied research protocol allowed for minimization of the potential influence of confounding factors, including maternal age, previous pregnancies, preterm delivery, obstetric emergencies, comorbid maternal or neonatal conditions, and medications used. It should be emphasized that the hormonal profile observed in the daughters participating in the present study differed substantially from that previously reported in daughters of women with hypothyroidism [12]. Reduced concentrations and shortened detection windows of LH and estradiol were accompanied by low progesterone levels, whereas no differences were observed in FSH concentrations. These findings warrant caution in interpreting euthyroid Hashimoto’s disease during pregnancy as a clinically insignificant or minimally significant laboratory or imaging abnormality, even if—as was the case in approximately half of the patients—it does not produce overt clinical symptoms. Furthermore, the results suggest that different maternal thyroid disorders may exert distinct effects on the course of minipuberty in their daughters. This supports the rationale for conducting analogous studies in the offspring of mothers with hyperthyroidism, goiter, and focal thyroid lesions. Finally, the potential long-term consequences of the observed alterations cannot be excluded, given that minipuberty represents the second of the three phases of activation of the reproductive axis [1]. It is postulated that one of its potential functions is to prepare the organism for the onset of true pubertal development, which typically occurs approximately a decade later [4]. Such an interpretation is supported by evidence demonstrating an association between increased activity of the hypothalamic–pituitary–ovarian axis during infancy in cases of low maternal vitamin D status [14] and earlier timing of individual stages of sexual maturation according to the Tanner scale [29].

The diagnosis of euthyroidism was based on the current recommendations of the American Thyroid Association [25]. In a recently published analysis of randomized clinical trials, hypothyroidism was also defined as a TSH concentration equal to or greater than 4.0 mU/L in the presence of normal free thyroxine levels [30]. It should be emphasized, however, that some authors have applied lower threshold values for the diagnosis of hypothyroidism during pregnancy (2.5 mU/L) [31,32]. These discrepancies prompted us to perform an additional analysis comparing gonadotropin, estradiol, and progesterone concentrations after excluding the offspring of women with a mean pregnancy TSH level exceeding 2.5 mU/L. This procedure, however, did not affect the mean concentrations or detection periods of the above hormones. Nevertheless, it cannot be entirely excluded that this finding may be related to the small number of mothers excluded from the analysis (n = 14), which is consistent with previous observations indicating that TSH follows a non-Gaussian, positively skewed distribution [33]. Therefore, the potential impact of mild thyroid dysfunction on the obtained results requires further investigation.

A clinically significant observation was the identification of differences in uterine and breast size between daughters of mothers with Hashimoto’s disease who did not receive supplementation and the offspring of women who received exogenous vitamin D and selenium, as well as the offspring of healthy women. Regardless of the study group, the size of both organs decreased between the first and second months of life, confirming earlier observations by another research team attributed to the postnatal withdrawal of the stimulatory effects of placental estrogens [10]. Those authors reported a reduction in the size of both structures in daughters of healthy, nonsmoking mothers from day 7 of life through 2–3 months of age; this decrease was more pronounced than in our study, which may be explained by the earlier initiation of follow-up in their cohort. Importantly, uterine length and breast diameter positively correlated with estrogen concentrations, whereas no analogous correlations were observed with antithyroid antibody titers. This finding may suggest that the smaller dimensions of the uterus and breasts are, at least in part, secondary to reduced activity of the reproductive axis. A second noteworthy observation was an additional decrease in uterine and breast size after the eighth month of life in the offspring of women with unsupplemented Hashimoto’s disease; this decrease was not observed in the remaining study groups. On the one hand, this finding indicates that the size of these structures is determined by estrogens produced by the infant herself rather than by estrogens of placental origin. On the other hand, this observation suggests that physiological development of the uterus and breast during minipuberty requires prolonged estrogen stimulation. Consequently, differences in the effects on these structures are most evident in the second half of minipuberty. The lack of continued follow-up during the period of hormonal quiescence after the completion of minipuberty precludes determination of whether these differences translate into variations in pubertal growth velocity or final adult size of these structures. Therefore, this issue warrants further investigation. Unlike daughters of women with untreated or poorly controlled hypothyroidism [12], female descendants of euthyroid women with Hashimoto’s disease exhibited a transient increase in ovarian volume between 2 and 10 months of age. This finding provides additional evidence for differences in the course of minipuberty between female offspring of women with autoimmune thyroiditis and those with hypothyroidism. The absence of differences in ovarian size between the study groups may be explained by an unchanged pattern of FSH secretion in daughters born to women with Hashimoto’s disease, as the concentration of this hormone was positively correlated with ovarian volume, whereas no similar correlations were observed for LH, estradiol, or progesterone. Despite comparable size, qualitative alterations in ovarian tissue resulting from local inflammation or oxidative stress cannot be ruled out [34]. Such changes would further support the use of vitamin D and selenium, both of which have been shown to exert inhibitory effects on these processes [35].

An analysis of intergroup comparisons and correlation patterns allows for the formulation of certain conclusions regarding the mechanisms underlying the observed alterations in the course of minipuberty. Although insufficient thyroid hormone action at the tissue level may be poorly reflected by serum measurements of TSH and free thyroid hormones, a potential link with tissue hypothyroidism does not appear fully plausible. This concept has been proposed to explain symptoms of hypothyroidism observed in some euthyroid individuals with autoimmune thyroiditis [36]. Moreover, differences in SPINA-GT—an index reflecting the thyroid gland’s maximum secretory capacity [37,38]—observed during pregnancy between both groups of mothers with Hashimoto’s disease may also align with the proposed mechanism. However, this parameter did not correlate with the concentrations of any of the evaluated hormones or with the size of the assessed organs. Thus, a more plausible explanation appears to be the impact of the autoimmune process itself. This interpretation is supported by the different dynamics of changes in hypothalamic–pituitary–gonadal axis hormone activity observed between the two groups of daughters born to mothers with autoimmune thyroiditis who differed, during pregnancy, in their TPOAb titers. Additionally, inverse correlations were identified between TPOAb titers and LH concentrations, as well as between LH and estradiol levels. These findings suggest that the effect of the autoimmune process on the reproductive axis in offspring may be centrally mediated, most likely at the level of the anterior pituitary gland. Such an interpretation seems to align with observations from other authors, who have reported that the placental barrier is permeable to antithyroid antibodies [39], and that the pituitary gland lies outside the blood–brain barrier, which permits the penetration of both antibodies and other inflammatory mediators into this structure [40]. The absence of similar correlations between LH concentrations and TgAb titers may be explained in two ways. First, TgAb demonstrate lower specificity and sensitivity for the diagnosis of Hashimoto’s disease compared with TPOAb [39]. Second, TgAb were assessed far less frequently in the mothers of our patients than TPOAb. A separate issue requiring further investigation is whether the observed effect of autoimmune thyroiditis results from thyroid autoimmunity per se, from concomitant autoimmune alterations within the placenta, or from a coexisting systemic inflammatory state associated with this disorder [41].

Particular attention should be given to the finding of lower progesterone concentrations in the group of daughters of women with Hashimoto’s disease who did not receive supplementation. Differences relative to the other groups were observed throughout the entire period of progesterone detection in saliva (12 months). To date, reduced progesterone concentrations have been reported exclusively in the offspring of women with inadequately controlled gestational diabetes [13]. The presence of inverse correlations between progesterone concentration and TPOAb titers suggests an association between decreased production and/or increased metabolism of progesterone and the severity of thyroid autoimmunity. However, the role of progesterone in infancy remains insufficiently understood, making it difficult to interpret the practical implications of the observed differences. One possible explanation is a potentially adverse effect on central nervous system development, as experimental studies suggest that this hormone may protect the developing brain and influence its maturation [42]. An alternative explanation involves a contributory role in the less pronounced growth of the uterus and mammary glands relative to daughters of healthy mothers. In adult women, progesterone modulates the effects of estrogens on estrogen-dependent tissues [43]. In line with this hypothesis, the correlations between estrogen concentrations and uterine and breast size were stronger in the group of women with Hashimoto’s disease who did not receive supplementation than in the other two groups. However, the absence of an association between progesterone concentration and the size of either structure argues against this interpretation. Finally, it cannot be excluded that the reduction in progesterone concentration represents an adaptive mechanism aimed at increasing diminished LH secretion, given that progesterone inhibits the pulsatile secretion of LH and lowers the LH-to-FSH ratio [44]. However, it remains unknown whether this effect also occurs during the developmental period.

The association between maternal disease during pregnancy and differences in the pattern of activation of the female reproductive axis during the first 18 months of life remains difficult to explain. It does not appear to be related to breastfeeding, as its frequency did not differ between the study groups. Nor can it be explained by differences in the baseline characteristics of the infants. Two alternative explanations may be considered more plausible, although both require further verification in future studies. The first explanation is based on the observation that minipuberty is preceded by an earlier phase of activation of the hypothalamic–pituitary–gonadal axis during fetal life, which in female fetuses begins in the second half of the first trimester [1]. In this context, maternal autoimmune disease during pregnancy may influence this initial phase of reproductive axis activation and thereby be associated with the differences observed during minipuberty. The second explanation relates to the concept of fetal programming, which refers to long-term adaptive changes in the developing fetus in response to unfavorable intrauterine environmental conditions [45]. This mechanism has previously been associated with chronic complications in the offspring of mothers with metabolic disorders and may also play a role in pregnancies complicated by thyroid disease [46].

The possibility of progression from subclinical hypothyroidism during pregnancy to overt hypothyroidism supports the rationale for levothyroxine use even in mild cases of autoimmune thyroid dysfunction [30,47]. Current guidelines of the American Thyroid Association also suggest considering levothyroxine therapy during pregnancy in women with autoimmune thyroiditis and TSH levels between 2.5 mIU/L and the upper limit of trimester-specific reference ranges (with higher levels requiring mandatory treatment) [25]. Therefore, levothyroxine use may have been considered in a small subgroup of mothers whose children were included in our study. We previously observed normalization of the course of minipuberty following reduction of TSH levels below 2.5 mIU/L; however, in the vast majority of mothers (including those with autoimmune hypothyroidism), baseline TSH levels exceeded 4.0 mIU/L [12,15]. Consequently, the impact of levothyroxine therapy on the course of minipuberty in offspring of women with TSH levels in the 2.5–4.0 mIU/L range remains unknown and warrants further investigation.

The present study is, however, the first to demonstrate the benefits of maternal administration of vitamin D and selenium during pregnancy, resulting in normalization of minipuberty in their daughters. The observed effect may support the rationale for using both micronutrients in euthyroid women with Hashimoto’s disease as an effective management option, especially when there is a desire to avoid thyroid hormone therapy. Unfortunately, the study protocol does not allow determination of the individual contribution of each micronutrient, nor does it exclude the possibility that the observed effect is attributable solely to one of the supplemented components, which is a significant limitation of the study. The observed negative correlations between reproductive axis activity and TPOAb titers suggest that the beneficial effects of vitamin D and selenium are, at least in part, a consequence of inhibition of the autoimmune process in the thyroid gland. Previous research by our team, which demonstrated a synergistic effect of these compounds on antithyroid antibody titers [48], further supports the notion that the observed effects result from the combined action of the supplemented micronutrients. Owing to the retrospective assessment of the mothers and the absence of such requirements in Polish guidelines, data on markers of vitamin D and selenium status during pregnancy were unavailable, except for retrospective questionnaire-based estimates of dietary intake. Nonetheless, dietary intake in the Polish population is lower than recommended [49], and similar findings were observed in mothers who did not use supplementation. Moreover, individuals with vitamin D deficiency are predisposed to developing autoimmune thyroiditis [20]. In prior studies, we observed that vitamin D deficiency in mothers without coexisting endocrine or immunological disorders was associated with higher urinary LH and FSH levels, as well as increased salivary estradiol, in their daughters [14]—representing a marked contrast to the present study. Additionally, vitamin D intake correlated with TPOAb titers but showed no association with evaluated hormones or measured organs. Therefore, it appears unlikely that the observed outcomes can be explained solely by normalization of vitamin D status. Given the lack of direct studies, the potential influence of the second supplemented component—selenium—cannot be excluded. Indirect evidence of its low supply, beyond the questionnaire analysis and prior studies conducted in the same geographic area [50], is provided by the low SPINA-GD index observed in the unsupplemented group. This parameter reflects the degree of endogenous thyroxine conversion to triiodothyronine [37,38], which is stimulated by selenoprotein-dependent deiodinases [51]. However, as with vitamin D, arguments against attributing the observed effects solely to selenium include the presence of a negative correlation between selenium intake and TPOAb titers, without corresponding correlations with reproductive axis activity or organ size. The beneficial effect of supplementation in our study is probably associated with the pharmacological impact of the studied micronutrients, as their intake was greater in the supplementation group than in the other two groups, which did not differ significantly.

The results obtained allow for several additional conclusions. First, mothers who received vitamin D and selenium supplementation during pregnancy exhibited lower mean TPOAb titers and higher mean SPINA-GT values, with a detectable correlation between these parameters. These findings seem to support the beneficial effects of the anti-inflammatory properties of vitamin D and selenium on thyroid secretory function. Second, full normalization of reproductive axis activity was observed despite the persistence of the thyroid autoimmune process, although its severity was reduced due to the additive effects of pregnancy and supplementation. This suggests the existence of a threshold between the intensity of the autoimmune process and its impact on LH, estradiol and progesterone secretion, or an enhanced anti-inflammatory effect mediated through additional mechanisms of vitamin D and selenium. Third, the period during which estradiol was detectable in saliva in daughters of healthy mothers (10 months) was twice as long as that of testosterone (5 months), despite testosterone being the primary source of estradiol through conversion [52]. This finding aligns with previous reports using different assay methods [1,2], making it unlikely that it reflects a lower detection threshold for estradiol compared with testosterone in saliva. It is more plausible that in infancy, most estradiol is produced almost exclusively via local conversion of testosterone in the ovaries. Fourth, no distinct peaks in gonadotropin or estradiol concentrations were observed in any of the studied groups, including daughters of healthy women. Previously reported fluctuations during longitudinal hormonal monitoring of individual girls were interpreted as resulting from periodic follicular growth and atresia [10]. The observed differences may reflect a lack of synchronization of these processes among different girls within the same group.

The study design (matched cohort study) inherently carries a risk of residual confounding and selection bias, which may have influenced the observed outcomes. Although randomization would reduce systematic bias, implementing such a design would have been challenging in this context. Initial contact with mothers occurred only after delivery, whereas randomization would need to be conducted during pregnancy, at an earlier stage of follow-up. Even if this logistical barrier were overcome, a randomized clinical trial could raise ethical concerns and potentially reduce participation rates. Some women would have been assigned to receive no vitamin D or selenium supplementation during pregnancy. Nevertheless, a randomized clinical trial conducted in the future would provide a robust comparison of the effects of vitamin D and selenium supplementation on the course of minipuberty in the offspring of mothers with autoimmune thyroiditis.

In addition to the previously mentioned limitations, several other constraints of the study should be noted. Although the sample size allowed for statistical analysis, the relatively small number of participants and recruitment from a single research center may limit the generalizability of the findings to broader populations; moreover, residual confounding cannot be excluded. Due to the relatively late enrollment of participants (during the second half of the first month of life), the study was unable to evaluate the hormonal dynamics characteristic of the earliest stage of minipuberty, which occurs within the first hours and days of life. While the immunoassays employed in this study are commonly used in routine practice, mass spectrometry-based techniques are more specific and are considered the gold standard for steroid quantification [53]. Despite the lack of differences in feeding modality, the potential influence of feeding practices on the results cannot be entirely excluded. Mandatory iodine prophylaxis in Poland, together with suboptimal selenium status among women living in Upper Silesia [50,54], suggests that the relationship between administration of vitamin D and selenium may differ from that observed in populations with limited iodine intake and/or adequate or excessive selenium intake. Despite efforts to address potential analytical biases, some influence of residual regression dilution and regression toward the mean on the results remains possible [55,56]. Finally, the study design does not permit investigation of the molecular and cellular mechanisms that may underlie differences in the course of minipuberty between daughters born to women with thyroiditis and those born to healthy women.

## 5. Conclusions

Daughters of euthyroid women with autoimmune thyroiditis, despite the absence of thyroid hormone substitution, exhibit lower concentrations of LH, estradiol, and progesterone during infancy, as well as a shorter duration of detectability for the first two hormones compared to daughters of healthy women. Differences in the dynamics of hypothalamic–pituitary–ovarian axis activity appear to depend on the severity of thyroid autoimmunity. Altered reproductive axis activity in the female offspring of women with thyroiditis is accompanied by smaller uterine and breast dimensions, while ovarian volume remains comparable to that observed in other girls of the same age. The course of female minipuberty does not differ from physiological patterns if, during pregnancy, women with Hashimoto’s disease receive vitamin D and selenium supplementation, although this intervention only partially mitigates thyroid autoimmunity. These findings support screening for autoimmune thyroiditis in all women with suspected disease, regardless of TSH and free thyroid hormone levels. They also provide evidence supporting the administration of vitamin D and selenium to euthyroid women of reproductive age with autoimmune thyroiditis. However, they do not allow determination of the independent contribution of each dietary micronutrient to the effects of their combined administration on female minipuberty. Given the pioneering nature of this study and the acknowledged limitations of the research protocol, confirmation of these findings in a well-designed, multicenter clinical trial involving a larger patient cohort is warranted.

## Figures and Tables

**Table 1:** Maternal characteristics of enrolled female infants (the intent-to-treat analysis).

Variable	Group A	Group B	Group C
Number (n)	32	32	32
Age (years)	31 ± 7	32 ± 8	32 ± 8
Primary or vocational/secondary/university education (%)	12/38/50	16/38/47	16/34/50
Occupational activity/white-collar/pink-collar/blue-collar workers (%)	88/32/40/16	91/28/47/16	88/32/44/12
Number of deliveries (n)	1.3 ± 0.6	1.4 ± 0.6	1.2 ± 0.5
Smokers (%)	19	16	16
Body mass index (kg/m^2^)	24.1 ± 4.0	23.8 ± 3.5	23.6 ± 3.2
Systolic blood pressure (mmHg)	121 ± 19	116 ± 17	118 ± 16
Diastolic blood pressure (mmHg)	80 ± 6	79 ± 6	80 ± 7
Total daily vitamin D intake (µg/day)	13.8 ± 4.0	64.2 ± 10.0 ^a^	20.8 ± 19.5
Total daily selenium intake (µg/day)	39 ± 14	120 ± 20 ^a^	50 ± 26

Data are presented as the mean and standard deviation, unless stated otherwise. Group A: Daughters of women with euthyroid autoimmune thyroiditis who did not receive vitamin D or selenium supplementation during pregnancy; Group B: Daughters of euthyroid women with autoimmune thyroiditis who received vitamin D and selenium supplementation during pregnancy; Group C: Daughters of healthy women without thyroid disorders during pregnancy. ^a^ *p* < 0.05 versus the other groups.

**Table 2:** Thyroid antibodies, hormones, and calculated parameters of thyroid homeostasis in mothers of female infants included in the study (the intent-to-treat analysis).

Variable	Group A	Group B	Group C
TPOAb [U/mL]			
Preconception	858 ± 294 ^b^ (n = 32)	895 ± 315 ^b^ (n = 32)	11 ± 10 (n = 24)
In pregnancy	545 ± 271 ^c^ (n = 32)	410 ± 168 ^a,c,d^ (n = 32)	12 ± 12 (n = 10)
TgAb [U/mL]			
Preconception	868 ± 401 ^b^ (n = 17)	847 ± 320 ^b^ (n = 21)	17 ± 12 (n = 15)
In pregnancy	625 ± 204 (n = 11)	425 ± 187 ^a,c,d^ (n = 12)	not assessed
TSH [mU/L]			
Preconception	2.1 ± 1.3 (n = 32)	2.1 ± 1.2 (n = 32)	1.5 ± 1.3 (n = 32)
In pregnancy	2.2 ± 1.1 (n = 32)	2.1 ± 1.0 (n = 32)	1.6 ± 1.3 (n = 32)
Free thyroxine [mU/L]			
Preconception	14.1 ± 2.8 (n = 32)	13.7± 3.5 (n = 32)	14.7 ± 4.0 (n = 8)
In pregnancy	14.0 ± 3.1 (n = 25)	16.0 ± 4.8 (n = 25)	not assessed
Free triiodothyronine [pmol/L]			
Preconception	3.1 ± 1.1 (n = 29)	3.0 ± 0.9 (n = 30)	not assessed
In pregnancy	3.2± 1.2 (n = 20)	3.8 ± 1.6 (n = 23)	not assessed
Jostel’s TSH index			
Preconception	2.6 ± 0.4 (n = 32)	2.6 ± 0.5 (n = 32)	2.4 ± 0.7 (n = 6)
In pregnancy	2.7 ± 0.5 (n = 25)	2.8 ± 0.5 (n = 25)	not assessed
SPINA-GT [pmol/s]			
Preconception	2.47 ± 0.85 (n = 32)	2.40 ± 0.75 (n = 32)	3.15 ± 1.25 (n = 6)
In pregnancy	2.39 ± 0.68 (n = 25)	2.81 ± 0.60 ^a,c,d^ (n = 25)	not assessed
SPINA-GD [nmol/s]			
Preconception	20.5± 2.6 (n = 29)	20.3 ± 3.0 (n = 30)	not assessed
In pregnancy	21.1 ± 2.4 (n = 20)	22.6 ± 2.3 ^a,c,d^ (n = 23)	not assessed

Data are presented as the mean and standard deviation, unless stated otherwise. Patient counts for each group at each time point are provided in parentheses. Group A: Daughters of women with euthyroid autoimmune thyroiditis who did not receive vitamin D or selenium supplementation during pregnancy; Group B: Daughters of euthyroid women with autoimmune thyroiditis who received vitamin D and selenium supplementation during pregnancy; Group C: Daughters of healthy women without thyroid disorders during pregnancy. ᵃ *p* < 0.05 versus group A; ^b^ *p* < 0.05 versus group C; ^c^ *p* < 0.05 versus the corresponding value before pregnancy; ^d^ *p* < 0.05—the percentage changes relative to the pre-pregnancy period are significantly larger in comparison to those in group A.

**Table 3:** Baseline features of infant participants (the intent-to-treat analysis).

Variable	Group A	Group B	Group C
Number (n)	32	32	32
Gestational age of delivery (weeks)	39 ± 2	39 ± 1	40 ± 1
Birth order: first/second/third and subsequent (%)	47/47/6	53/38/9	50/44/6
Length (cm)	53.9 ± 1.8	54.1 ± 1.6	54.4 ± 1.9
Weight (kg)	4.35 ± 0.58	4.51 ± 0.53	4.432 ± 0.61
Weight-for-length percentile	69 ± 16	71 ± 15	64 ± 18
Head circumference (cm)	36.9 ± 0.9	36.8 ± 0.9	37.1 ± 0.8
Breastfeeding (%)	75	78	81
Total daily vitamin D intake (µg)	13.5 ± 1.5	13.2 ± 1.6	12.9 ± 1.5
Total daily selenium intake (µg)	10.7 ± 1.2	11.0 ± 1.2	11.2 ± 1.1

Data are presented as the mean and standard deviation, unless stated otherwise. Group A: Daughters of women with euthyroid autoimmune thyroiditis who did not receive vitamin D or selenium supplementation during pregnancy; Group B: Daughters of euthyroid women with autoimmune thyroiditis who received vitamin D and selenium supplementation during pregnancy; Group C: Daughters of healthy women without thyroid disorders during pregnancy.

**Table 4:** Associations between the assessed variables.

Correlated Variables		Group A	Group B	Group C
LH	Estradiol	0.50 [*p* < 0.0001]–0.62 [*p* < 0.0001]	0.47 [*p* = 0.0001]–0.65 [*p* < 0.0001]	0.46 [*p* = 0.0002]–0.68 [*p* < 0.0001]
Gestational TPOAb	Total vitamin D intake during pregnancy	−0.46 [*p* = 0.0002]	−0.51 [*p* < 0.0001]	not assessed
Gestational TPOAb	Total selenium intake during pregnancy	−0.38 [*p* = 0.0058]	−0.46 [*p* = 0.0001]	not assessed
Gestational TPOAb	Gestational TSH	0.42 [*p* = 0.0008]	0.39 [*p* = 0.0029]	not assessed
Gestational TPOAb	LH	−0.44 [*p* = 0.0004]–−0.62 [*p* < 0.0001]	−0.40 [*p* = 0.0012]–−0.61 [*p* < 0.0001]	not assessed
Gestational TPOAb	Progesterone	−0.31 [*p* = 0.0400]–−0.41 [*p* = 0.0008]	−0.29 [*p* = 0.0461]–−0.43 [*p* = 0.0007]	not assessed
Gestational TPOAb	Gestational SPINA-GT	−0.28 [*p* = 0.0482]	−0.42 [*p* = 0.0008]	not assessed
FSH	Ovarian volume	0.29 [*p* = 0.0465]–0.42 [*p* = 0.0007]	0.28 [*p* = 0.0470]–0.40 [*p* = 0.0014]	0.32 [*p* = 0.0298]–0.46 [*p* = 0.0001]
Estradiol	Uterine length	0.43 [*p* = 0.0006]–0.56 [*p* < 0.0001]	0.35 [*p* = 0.0163]–0.44 [*p* = 0.0005]	0.36 [*p* = 0.0116]–0.46 [*p* = 0.0001]
Estradiol	Breast diameter	0.44 [*p* = 0.0112]–0.59 [*p* < 0.0001]	0.36 [*p* = 0.0096]–0.46 [*p* = 0.0002]	0.34 [*p* = 0.0235]–0.44 [*p* = 0.0006]

The data show the correlation coefficients (r values). Group A: Daughters of women with euthyroid autoimmune thyroiditis who did not receive vitamin D or selenium supplementation during pregnancy; Group B: Daughters of euthyroid women with autoimmune thyroiditis who received vitamin D and selenium supplementation during pregnancy; Group C: Daughters of healthy women without thyroid disorders during pregnancy.

## Data Availability

The data presented in this study are available upon request from the corresponding author due to privacy restrictions.

## Supplementary Materials

The following supporting information can be downloaded at: https://www.mdpi.com/article/10.3390/nu18121841/s1.
