# Supplementary material for: Impact of Maternal Euthyroid Autoimmune Thyroiditis on Minipuberty in Female Offspring
Source: Nutrients. 2026 Jun 7;18(12):1841. doi: 10.3390/nu18121841 (PMC13304813; doi:10.3390/nu18121841)
Supplement: Supplementary file 1 [file nutrients-18-01841-s001.zip › nutrients-4328228-supplementary.pdf]

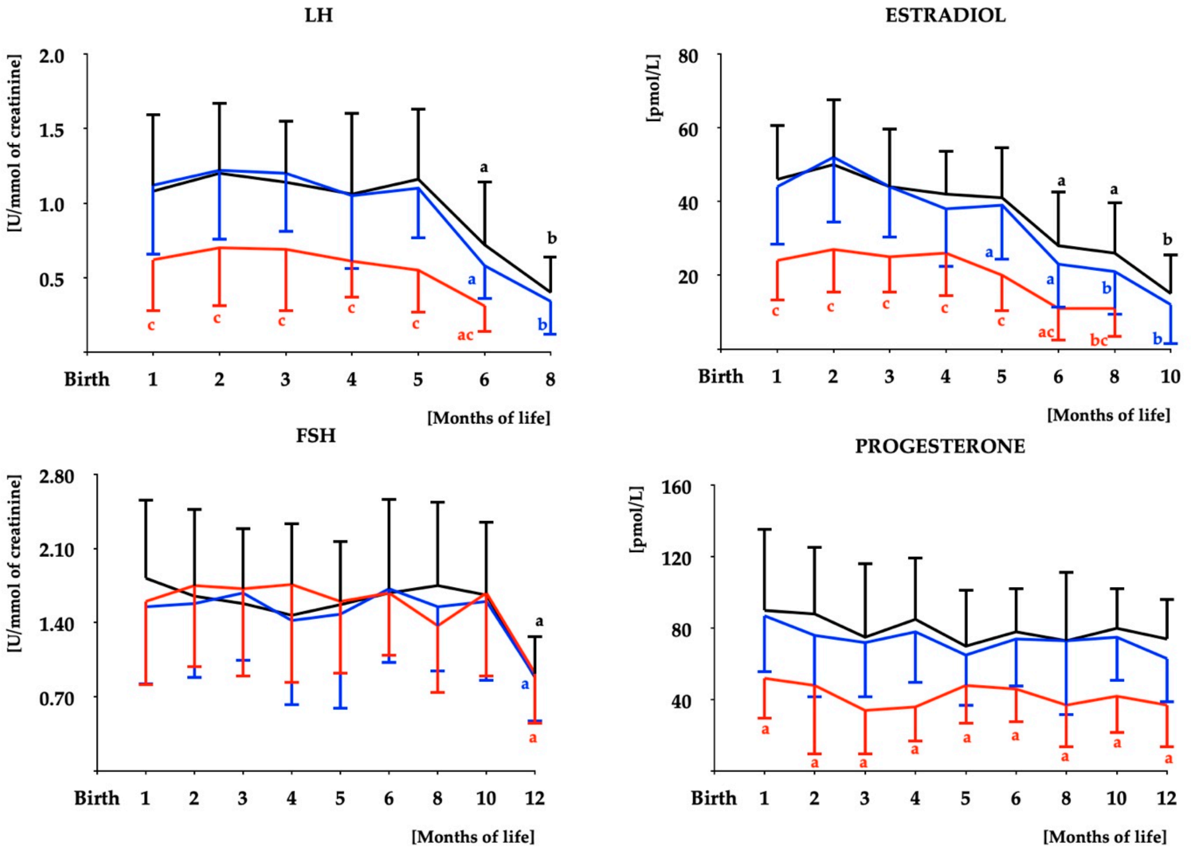

Supplementary Figure S1. Concentrations of gonadotropins, estradiol, and progesterone in offspring of mothers with mean pregnancy TSH ≤ 2.5 mU/L

Data are expressed as mean ± standard deviation. LH was undetectable in urine from month 8 until the end of the study in group A and from month 10 until the end of the study in groups B and C. FSH was undetectable in urine from month 15 until the end of the study. Estradiol was undetectable in saliva from month 10 in group A and from month 12 in groups B and C until the end of the study. Progesterone was undetectable in saliva from month 15 until the end of the study. Group A (red line): Daughters of women with euthyroid autoimmune thyroiditis who did not receive vitamin D or selenium supplementation during pregnancy; Group B (blue line): Daughters of euthyroid women with autoimmune thyroiditis who received vitamin D and selenium supplementation during pregnancy; Group C (black line): Daughters of healthy women without thyroid disorders during pregnancy. Statistical annotations: LH - $^{a}p < 0.05$ versus months 1-5 within the same study group, $^{b}p < 0.05$ versus months 1–6 within the same study group, $^{c}p < 0.05$ versus the corresponding time point in the other groups; FSH - $^{a}p < 0.05$ versus months 1-10 within the same study group; estradiol - $^{a}p < 0.05$ versus months 1-5 within the same study group, $^{b}p < 0.05$ versus months 1–8 within the same study group, $^{c}p < 0.05$ versus the corresponding time point in the other groups; progesterone - $^{a}p < 0.05$ versus the corresponding time point in the other groups.
